# Supplementary material for: A gap-free, telomere-to-telomere genome assembly for the Caenorhabditis briggsae reference strain AF16
Source: G3 (Bethesda). 2026 Jun 18;16(8):jkag158. doi: 10.1093/g3journal/jkag158 (PMC13439942; doi:10.1093/g3journal/jkag158)
Supplement: Supplementary file 1 [file jkag158_supplementary_data.zip › Supplementary_Figure_10_G3-2026-406856.pdf]

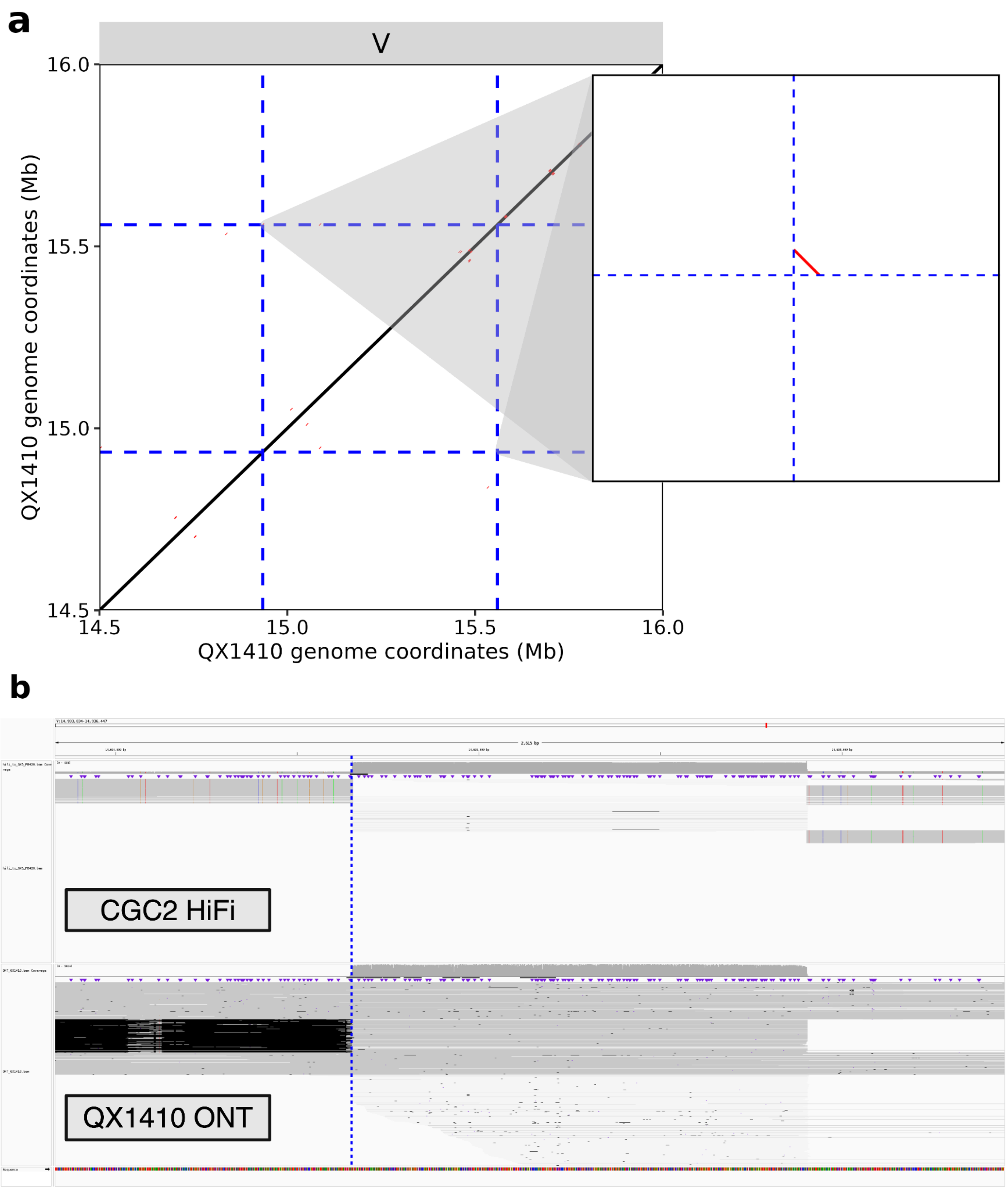


**Supplementary Figure 10.** A private QX1410 inversion allele. a) Whole-genome alignments of QX1410 to itself at the locus that is inverted in other *C. briggsae* reference genomes (Supplementary Figure 9). Inset displays multi-aligning sequence flanking the inversion breakpoints (dashed blue lines) b) IGV screenshot of CGC2 PacBio HiFi read alignments (first row) and QX1410 ONT read alignments (second row) to the QX1410 genome over the left inversion breakpoint in QX1410 (vertical dashed blue line). High-quality read mappings are shaded in grey.
