# Supplementary material for: A gap-free, telomere-to-telomere genome assembly for the Caenorhabditis briggsae reference strain AF16
Source: G3 (Bethesda). 2026 Jun 18;16(8):jkag158. doi: 10.1093/g3journal/jkag158 (PMC13439942; doi:10.1093/g3journal/jkag158)
Supplement: Supplementary file 1 [file jkag158_supplementary_data.zip › Supplementary_Figure_11_G3-2026-406856.pdf]

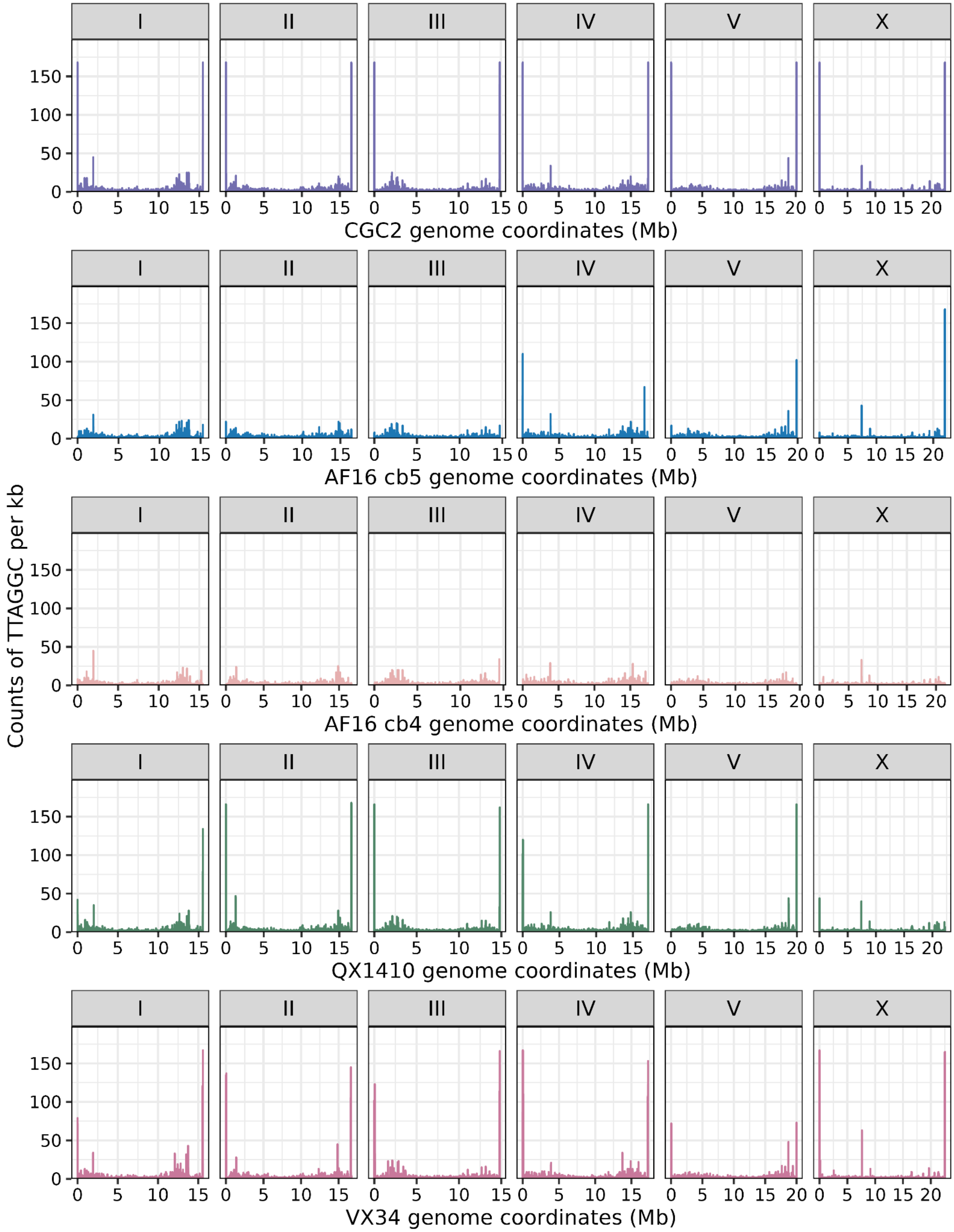

Supplementary Figure 11. Telomeric repeat sequence in *C. briggsae* genome assemblies. Counts of nematode telomere repeat sequence (TTAGGC) per kb bin in *C. briggsae* chromosome-level genome assemblies. Counts of 167 represent telomeric repeat sequence spanning an entire kb window.
