# Supplementary material for: A gap-free, telomere-to-telomere genome assembly for the Caenorhabditis briggsae reference strain AF16
Source: G3 (Bethesda). 2026 Jun 18;16(8):jkag158. doi: 10.1093/g3journal/jkag158 (PMC13439942; doi:10.1093/g3journal/jkag158)
Supplement: Supplementary file 1 [file jkag158_supplementary_data.zip › Supplementary_Figure_12_G3-2026-406856.pdf]

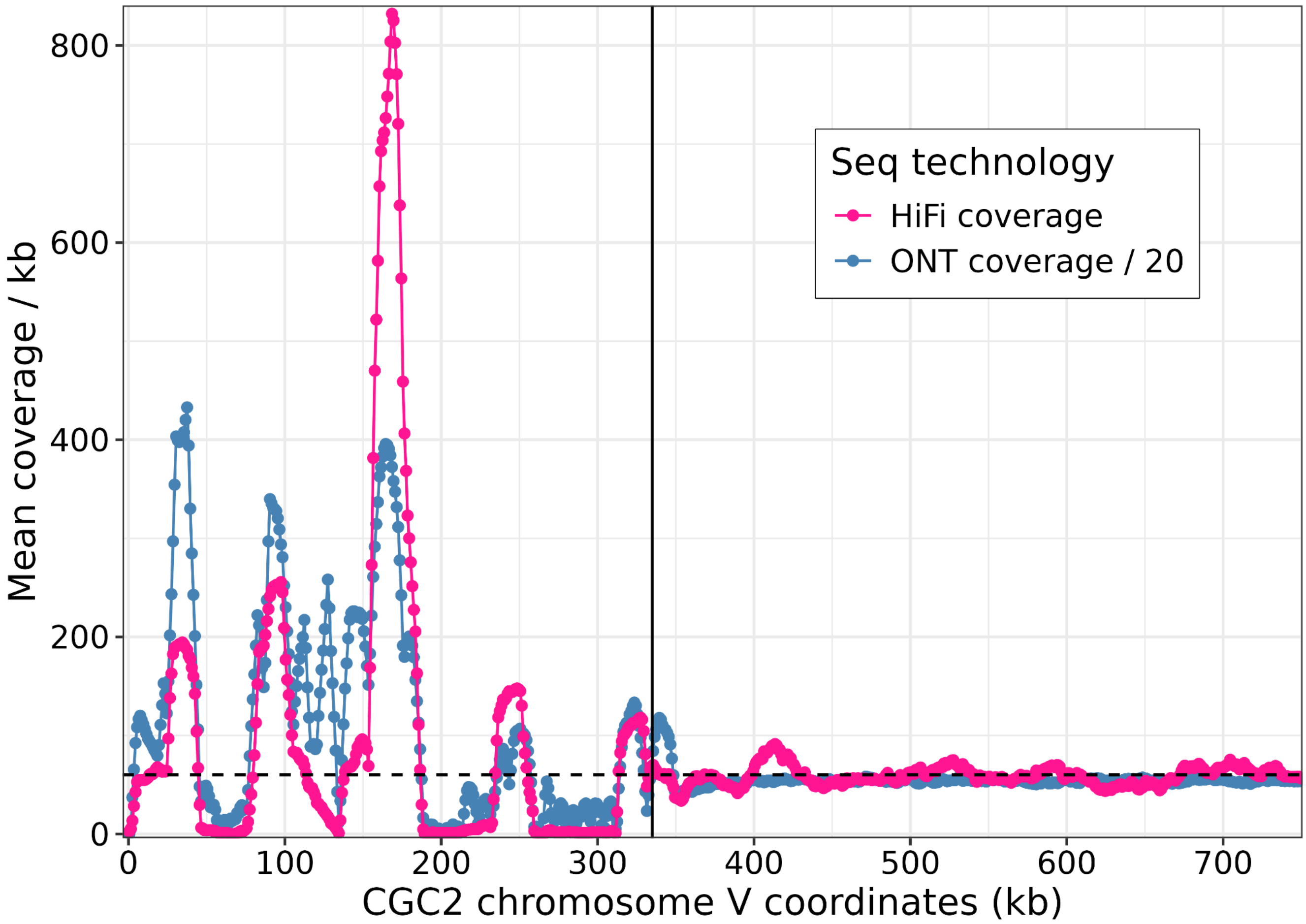

Supplementary Figure 12. Long-read sequencing coverage on the left-end of CGC2 chromosome V. HiFi and ONT read coverage over telomeric and rDNA repeat regions (0 kb - 335 kb (vertical black line)).
