# Supplementary material for: A gap-free, telomere-to-telomere genome assembly for the Caenorhabditis briggsae reference strain AF16
Source: G3 (Bethesda). 2026 Jun 18;16(8):jkag158. doi: 10.1093/g3journal/jkag158 (PMC13439942; doi:10.1093/g3journal/jkag158)
Supplement: Supplementary file 1 [file jkag158_supplementary_data.zip › Supplementary_Figure_13_G3-2026-406856.pdf]

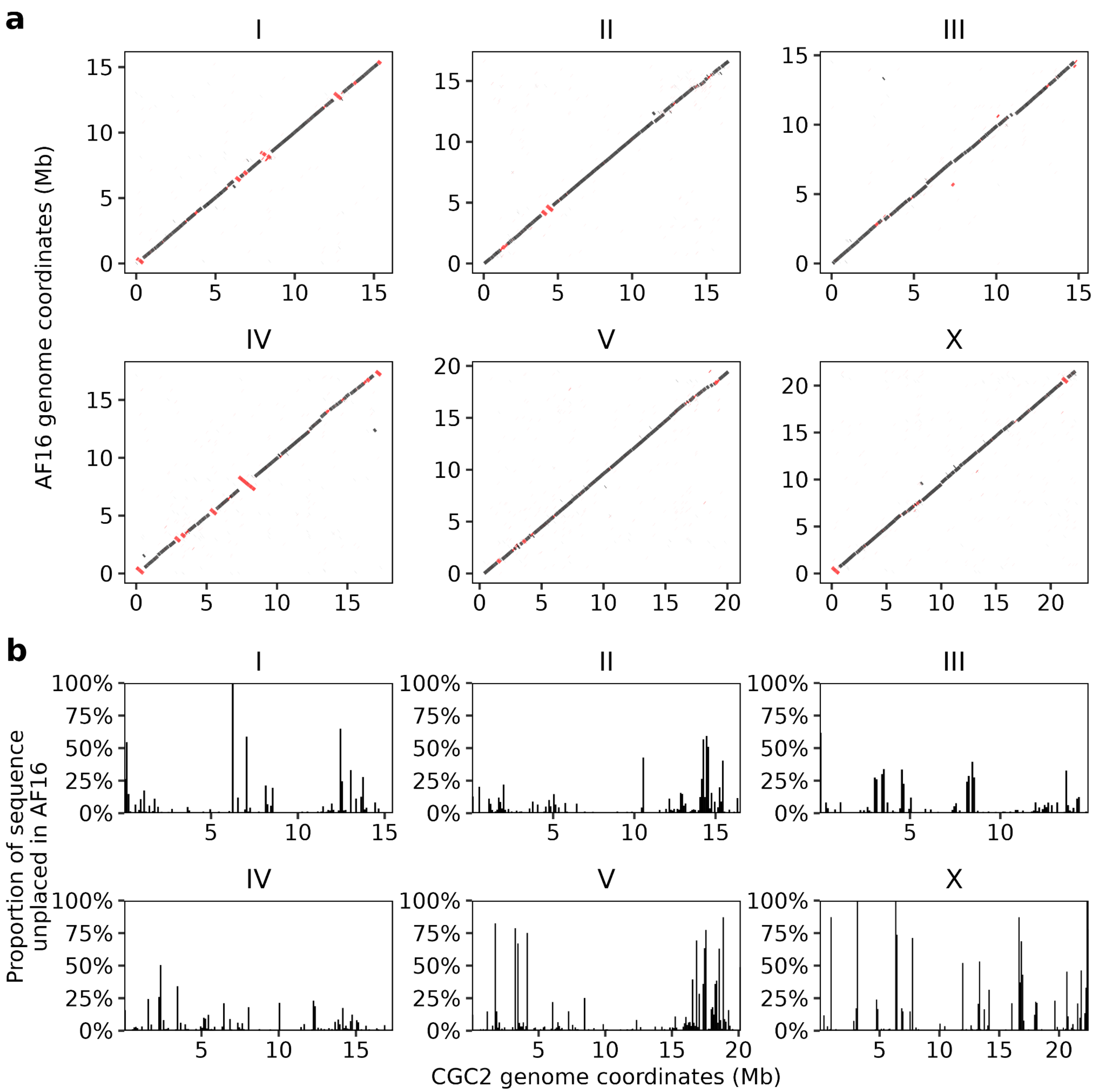


**Supplementary Figure 13.** Whole-genome alignment of AF16 cb4 to CGC2. (a) Colinearity of AF16 cb4 to CGC2 genomes generated using nucmer. Inter-chromosomal alignments and alignments spanning less than 1 kb are not shown. Alignments in the reverse orientation are displayed in red. (b) The proportion of sequence that is contained in AF16 cb4 unplaced scaffolds per 100 kb window across the CGC2 genome.
