# Supplementary material for: A gap-free, telomere-to-telomere genome assembly for the Caenorhabditis briggsae reference strain AF16
Source: G3 (Bethesda). 2026 Jun 18;16(8):jkag158. doi: 10.1093/g3journal/jkag158 (PMC13439942; doi:10.1093/g3journal/jkag158)
Supplement: Supplementary file 1 [file jkag158_supplementary_data.zip › Supplementary_Figure_14_G3-2026-406856.pdf]

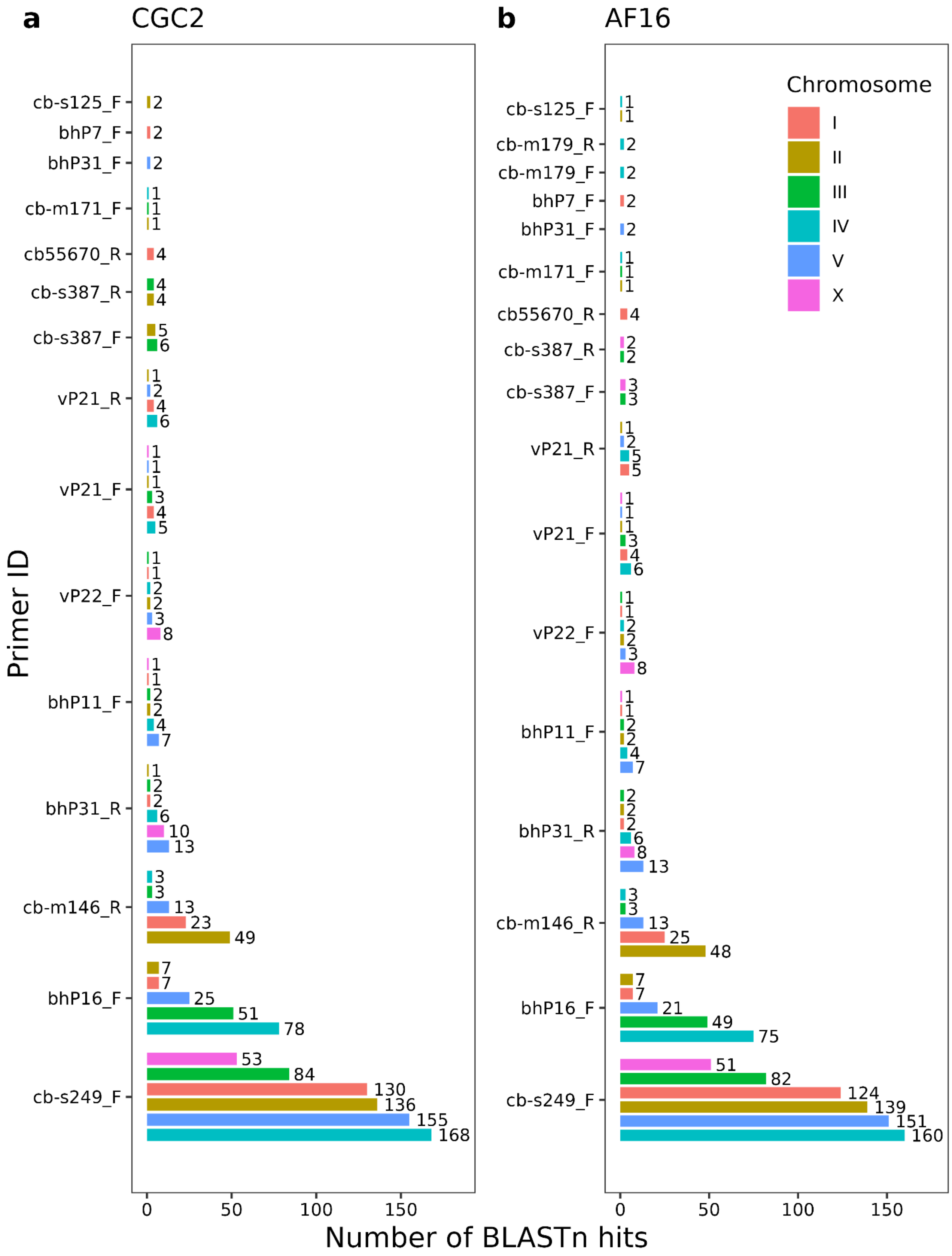

Supplementary Figure 14. Multi-mapping insertion-deletion primers. Bar charts showing the number of BLASTn hits (x-axis) for each primer (y-axis) with more than two mapping locations in the (a) CGC2 genome or (b) AF16 cb4 genome. Primers with multiple bars indicate that it was mapped to multiple chromosomes, with bars colored by chromosome ID.
