# Supplementary material for: A gap-free, telomere-to-telomere genome assembly for the Caenorhabditis briggsae reference strain AF16
Source: G3 (Bethesda). 2026 Jun 18;16(8):jkag158. doi: 10.1093/g3journal/jkag158 (PMC13439942; doi:10.1093/g3journal/jkag158)
Supplement: Supplementary file 1 [file jkag158_supplementary_data.zip › Supplementary_Figure_15_G3-2026-406856.pdf]

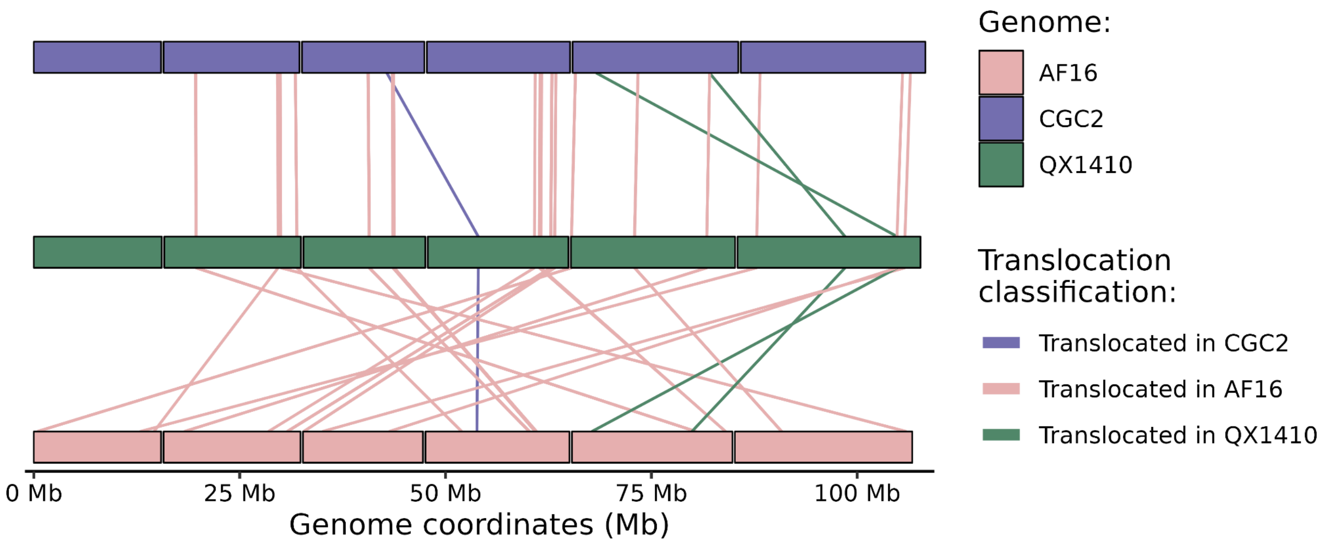

Supplementary Figure 15. Inter-chromosomal single-copy ortholog mappings between *C. briggsae* genomes. Each bar represents a *C. briggsae* chromosome colored and organized in rows by reference genome (CGC2, purple; QX1410, green; AF16 cb4, pink). Genome position in megabases is displayed on the x-axis. Lines represent the positions of single-copy ortholog mappings across any two genomes. Lines colored in pink represent single-copy orthologs that map within the same chromosome in CGC2 and QX1410 but map to a different chromosome in AF1 cb46. Lines colored in green represent single-copy orthologs that map within the same chromosome in CGC2 and AF16 cb4 but map across to a different chromosome in QX1410. Lines colored in purple represent single-copy orthologs that map within the same chromosome in QX1410 and AF16 cb4 but map to a different chromosome in CGC2.
