# Supplementary material for: A gap-free, telomere-to-telomere genome assembly for the Caenorhabditis briggsae reference strain AF16
Source: G3 (Bethesda). 2026 Jun 18;16(8):jkag158. doi: 10.1093/g3journal/jkag158 (PMC13439942; doi:10.1093/g3journal/jkag158)
Supplement: Supplementary file 1 [file jkag158_supplementary_data.zip › Supplementary_Figure_16_G3-2026-406856.pdf]

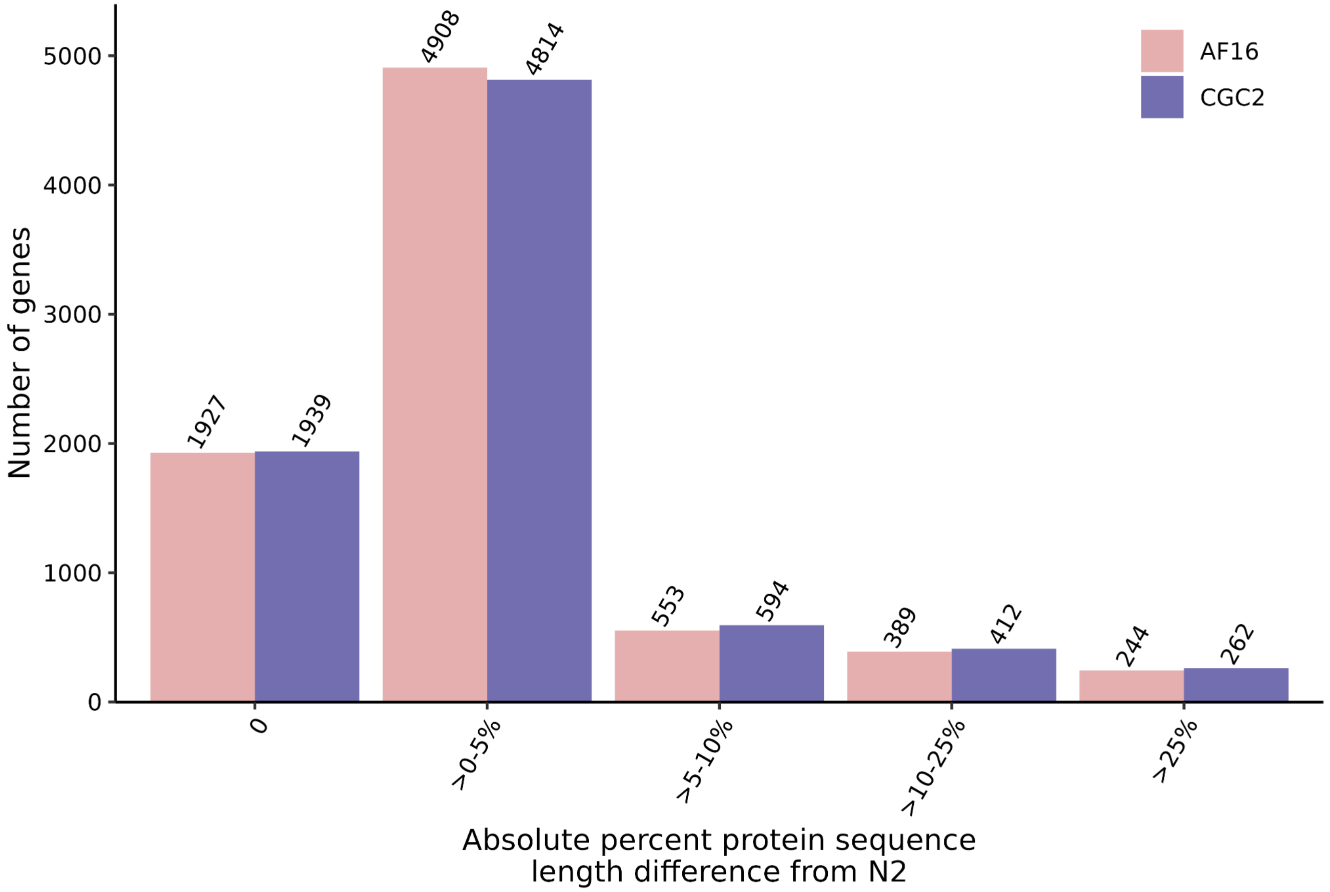

Supplementary Figure 16. Comparison of protein length accuracy of *C. briggsae* single-copy orthologs. Bar charts showing the number of single-copy orthologs between *C. briggsae* gene annotations (AF16 cb4, pink; CGC2, purple) across different bins of percent protein sequence length difference from single-copy *C. elegans* N2 orthologs. Identical protein sequences between *C. briggsae* and *C. elegans* will have a percent difference of zero, increasing in value depending on how much shorter or longer the *C. briggsae* protein sequence is from its N2 ortholog. Only single-copy orthologs present across all three AF16 cb4, CGC2, and N2 are included.
