# Supplementary material for: A gap-free, telomere-to-telomere genome assembly for the Caenorhabditis briggsae reference strain AF16
Source: G3 (Bethesda). 2026 Jun 18;16(8):jkag158. doi: 10.1093/g3journal/jkag158 (PMC13439942; doi:10.1093/g3journal/jkag158)
Supplement: Supplementary file 1 [file jkag158_supplementary_data.zip › Supplementary_Figure_1_G3-2026-406856.pdf]

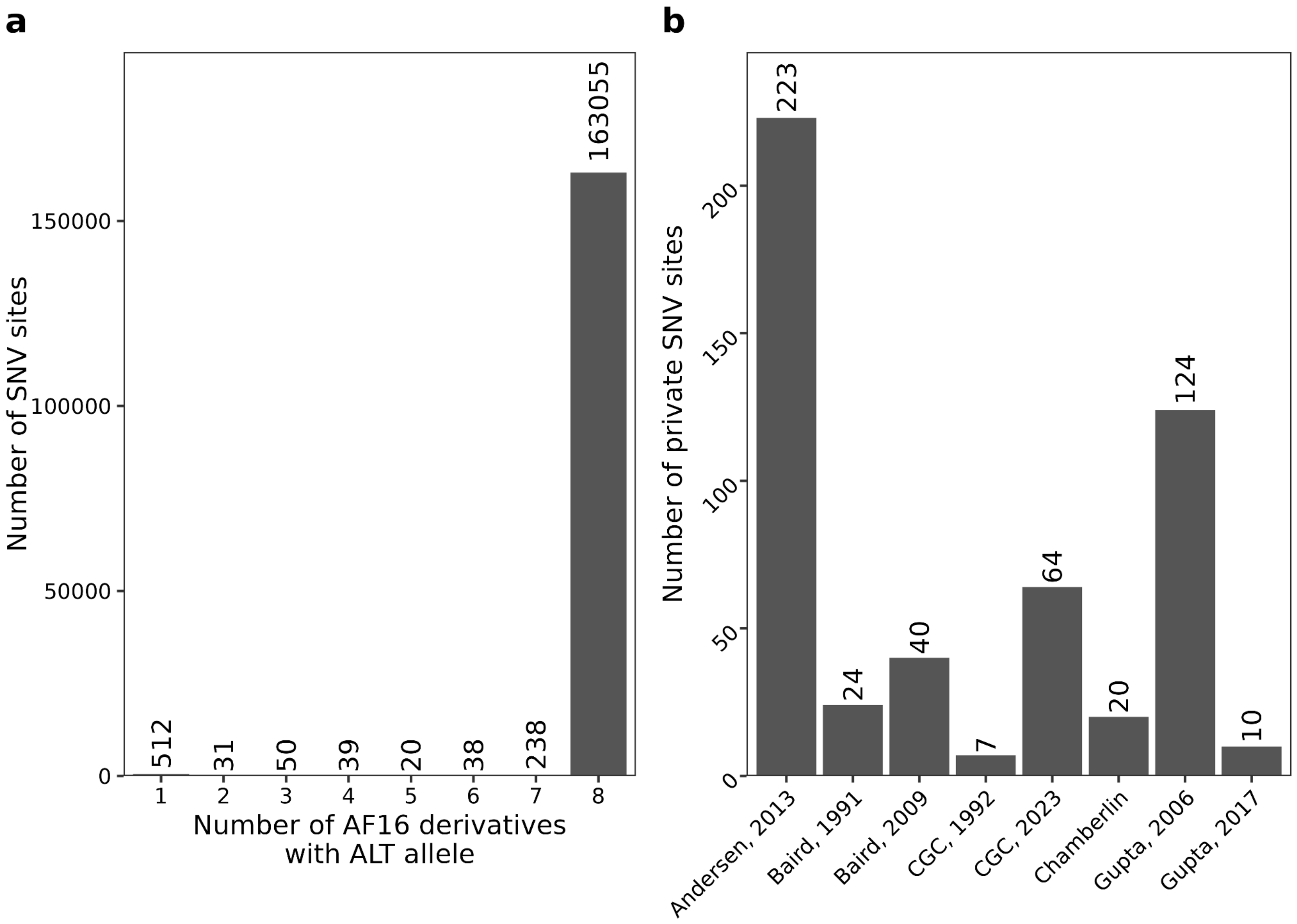

Supplementary Figure 1. Alternative alleles across AF16 derivatives. Only SNV sites with perfect information (no missing genotypes) are considered. (a) Bar chart showing the number of SNV sites where one or more AF16 derivative strains carry an alternative (ALT) allele relative to the QX1410 reference genome. All eight AF16 derivatives carry an ALT allele at the vast majority (163,055) of SNV sites, representing natural variants between the AF16 derivatives and QX1410. A total of 512 sites are private to only one AF16 derivative. (b) Bar chart showing the number of ALT alleles at private SNV sites across AF16 derivative strains (labels display source laboratory and the year that the strain was cryopreserved.
