# Supplementary material for: A gap-free, telomere-to-telomere genome assembly for the Caenorhabditis briggsae reference strain AF16
Source: G3 (Bethesda). 2026 Jun 18;16(8):jkag158. doi: 10.1093/g3journal/jkag158 (PMC13439942; doi:10.1093/g3journal/jkag158)
Supplement: Supplementary file 1 [file jkag158_supplementary_data.zip › Supplementary_Figure_2_G3-2026-406856.pdf]

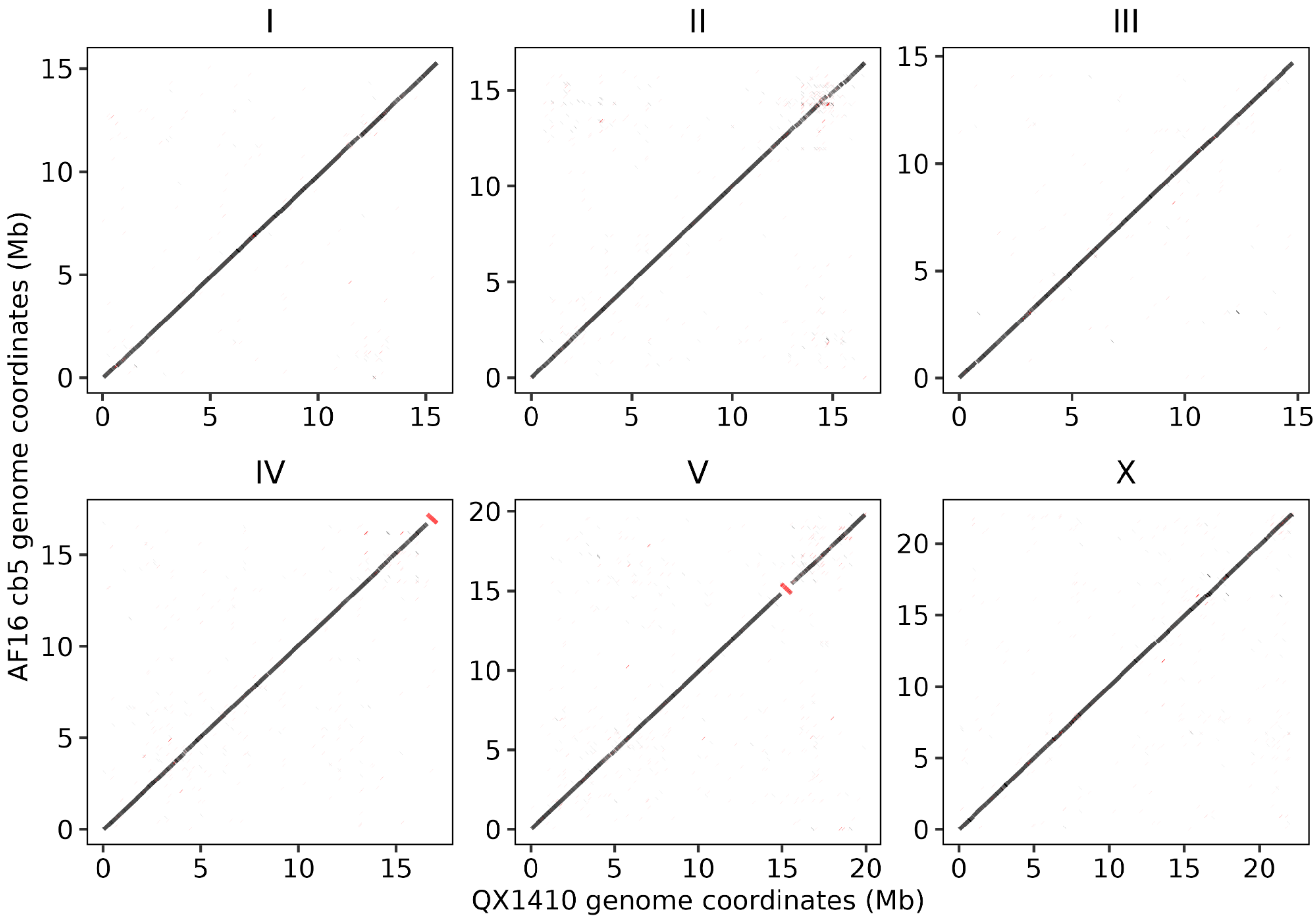

Supplementary Figure 2. Whole-genome alignment of AF16 cb5 to QX1410. Colinearity of AF16 cb5 to QX1410 genomes generated using nucmer. Inter-chromosomal alignments and alignments spanning less than 1 kb are not shown. Alignments in the reverse orientation are displayed in red.
