# Supplementary material for: A gap-free, telomere-to-telomere genome assembly for the Caenorhabditis briggsae reference strain AF16
Source: G3 (Bethesda). 2026 Jun 18;16(8):jkag158. doi: 10.1093/g3journal/jkag158 (PMC13439942; doi:10.1093/g3journal/jkag158)
Supplement: Supplementary file 1 [file jkag158_supplementary_data.zip › Supplementary_Figure_3_G3-2026-406856.pdf]

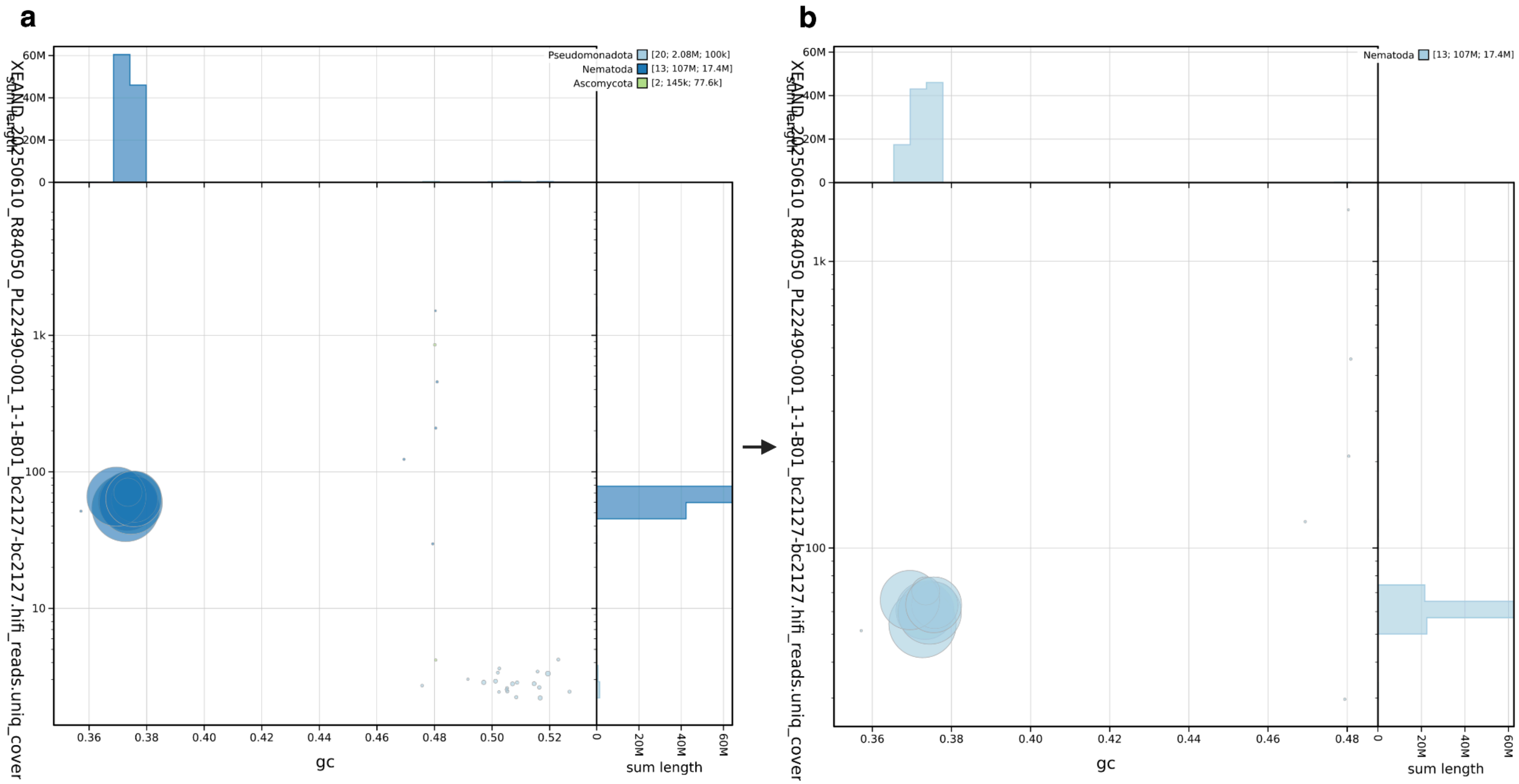

Supplementary Figure 3. Blob plots of CGC2 contig taxonomy classification. (a) A blob plot produced using BlobToolKit. Contigs are colored by their taxonomic classification. The x-axis displays contig GC content, and the y-axis displays HiFi read coverage of contigs. (b) The resulting blob plot of 13 contigs after removing contigs that were not classified as *Nematoda*.
