# Supplementary material for: A gap-free, telomere-to-telomere genome assembly for the Caenorhabditis briggsae reference strain AF16
Source: G3 (Bethesda). 2026 Jun 18;16(8):jkag158. doi: 10.1093/g3journal/jkag158 (PMC13439942; doi:10.1093/g3journal/jkag158)
Supplement: Supplementary file 1 [file jkag158_supplementary_data.zip › Supplementary_Figure_4_G3-2026-406856.pdf]

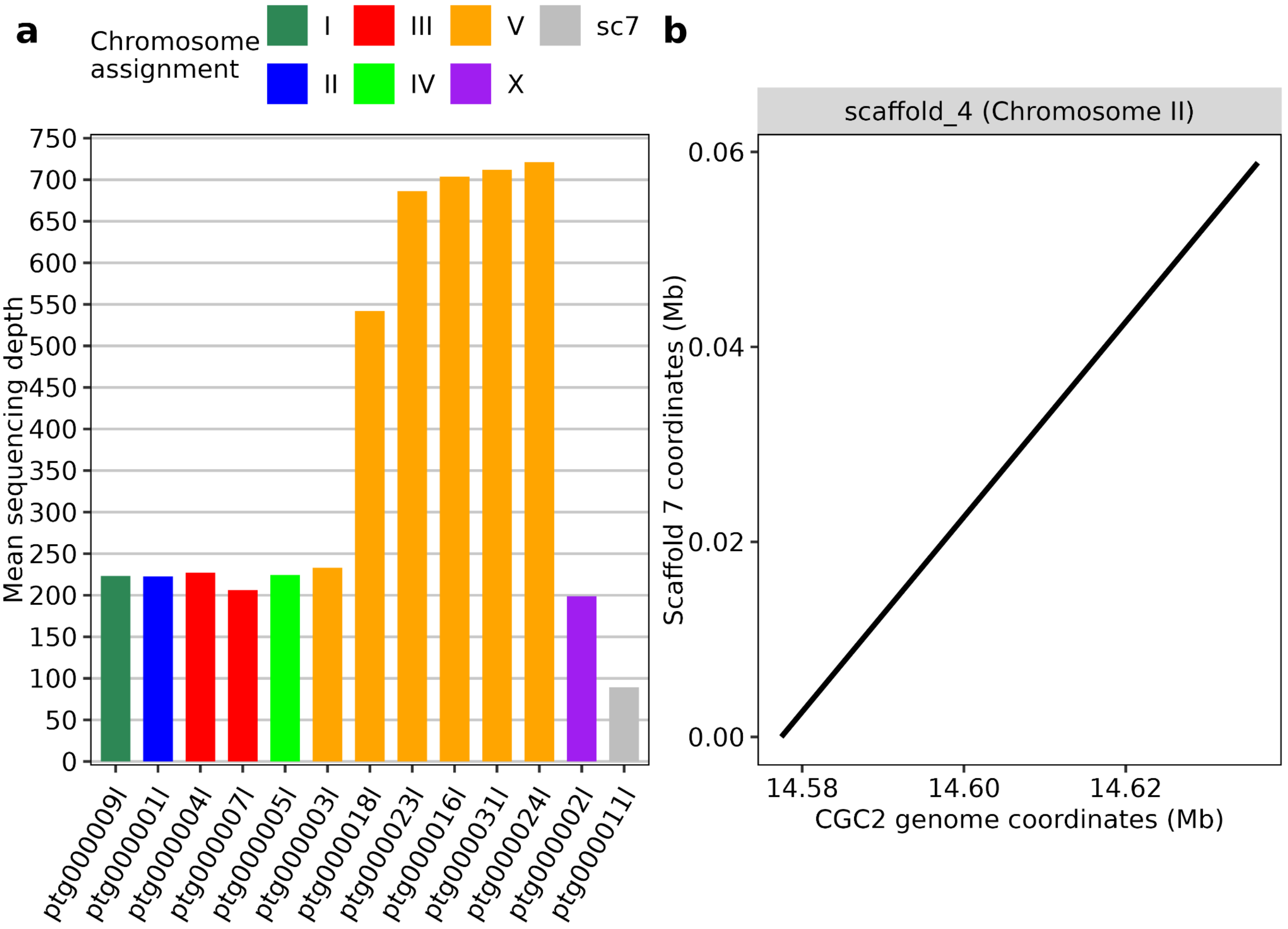

Supplementary Figure 4. Identification of the duplicate haplotig, scaffold 7. (a) Hi-C read fold-coverage of the 13 contigs used in scaffolding. Contigs are colored by their final chromosome identification after scaffolding, with scaffold 7 (sc7) corresponding to a single contig. (b) Alignment of scaffold 7 to CGC2 scaffolds, where scaffold 7 aligns to a region in scaffold 4 (chromosome II).
