# Supplementary material for: A gap-free, telomere-to-telomere genome assembly for the Caenorhabditis briggsae reference strain AF16
Source: G3 (Bethesda). 2026 Jun 18;16(8):jkag158. doi: 10.1093/g3journal/jkag158 (PMC13439942; doi:10.1093/g3journal/jkag158)
Supplement: Supplementary file 1 [file jkag158_supplementary_data.zip › Supplementary_Figure_5_G3-2026-406856.pdf]

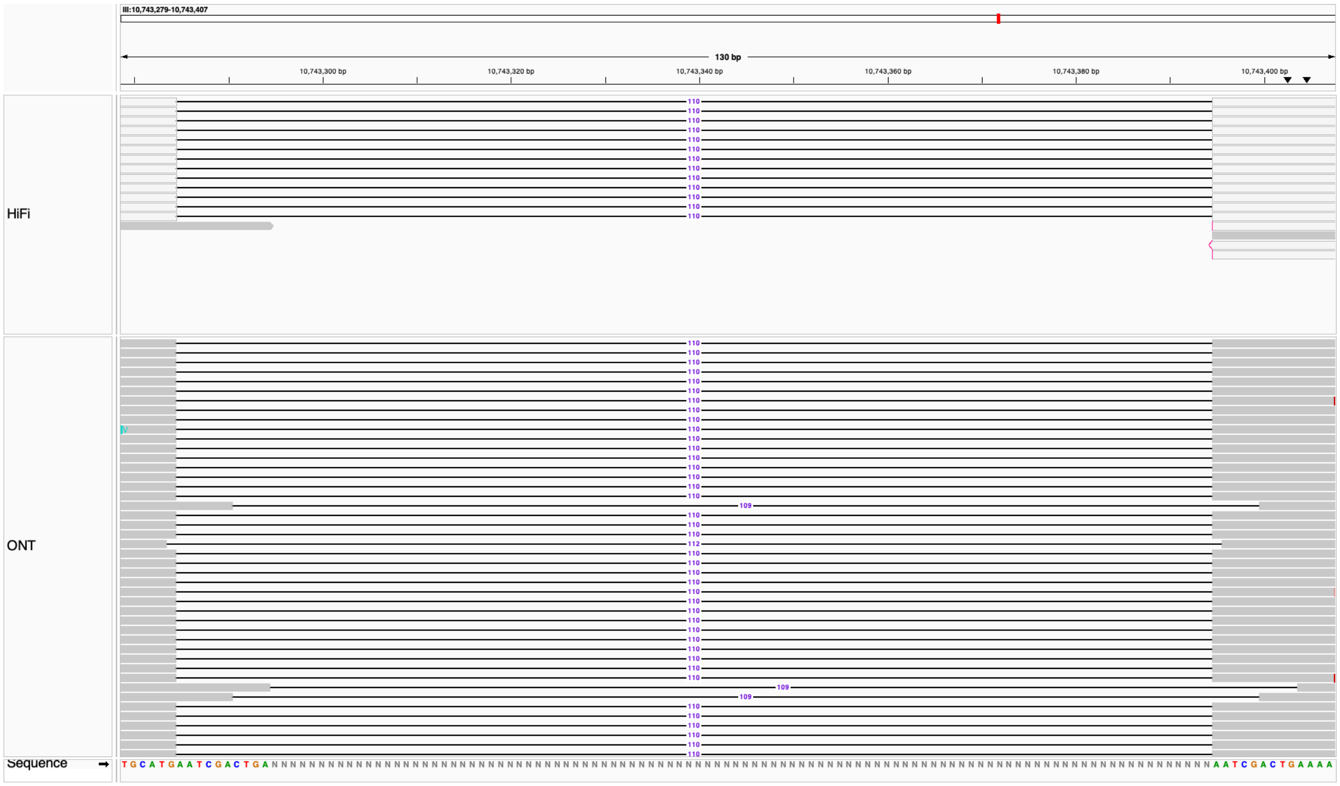

Supplementary Figure 5. Long-read sequencing reads alignments to CGC2 chromosome III gap in IGV. The first row displays PacBio HiFi read alignments and the second row displays ONT read alignments. The gap is represented with 100 Ns, displayed in the bottom sequence panel, where the last 100 bps upstream of the gap and the first 100 bps downstream of the gap are identical. The removal of the 100 Ns and the 10 bps upstream of the gap is supported by multi-mapping HiFi reads and MAPQ60 ONT reads. ONT reads are sorted in descending order of mapping quality and grey-filled reads indicate primary read alignments.
