# Supplementary material for: A gap-free, telomere-to-telomere genome assembly for the Caenorhabditis briggsae reference strain AF16
Source: G3 (Bethesda). 2026 Jun 18;16(8):jkag158. doi: 10.1093/g3journal/jkag158 (PMC13439942; doi:10.1093/g3journal/jkag158)
Supplement: Supplementary file 1 [file jkag158_supplementary_data.zip › Supplementary_Figure_6_G3-2026-406856.pdf]

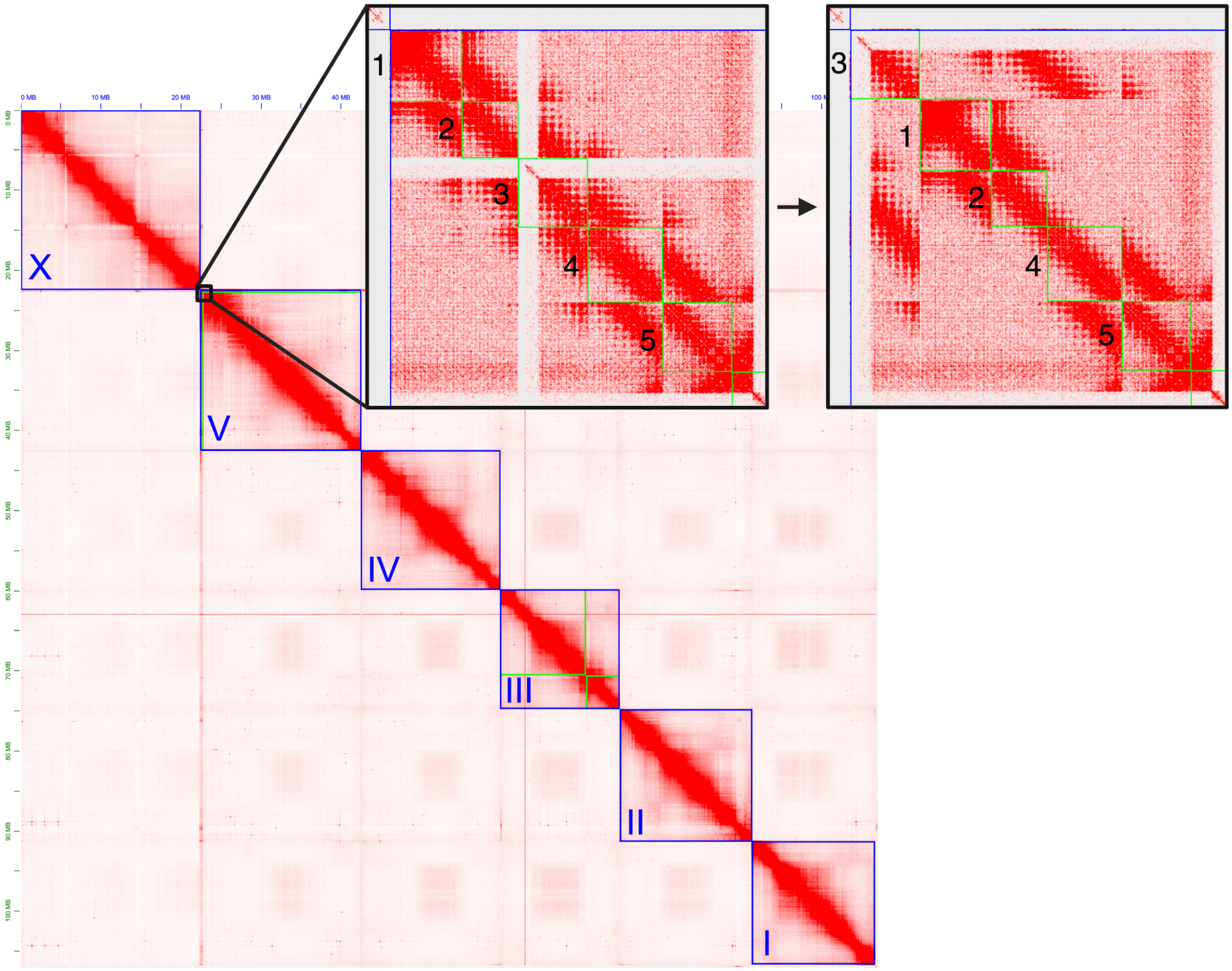

Supplementary Figure 6. Hi-C contact map of CGC2 genome assembly. The diagonals inside blue squares represent nuclear chromosomes and the diagonals inside smaller, green squares represent contigs. Chromosome III is constructed from two contigs, where chromosome V is constructed from six contigs. The left inset displays five labeled contigs at the left-end of chromosome V, where five gaps are present (vertices of green squares). The right inset displays the resulting contact map after moving contig three, which contains telomeric repeat sequence, to the terminal left-end of chromosome V.
