# Supplementary material for: A gap-free, telomere-to-telomere genome assembly for the Caenorhabditis briggsae reference strain AF16
Source: G3 (Bethesda). 2026 Jun 18;16(8):jkag158. doi: 10.1093/g3journal/jkag158 (PMC13439942; doi:10.1093/g3journal/jkag158)
Supplement: Supplementary file 1 [file jkag158_supplementary_data.zip › Supplementary_Figure_7_G3-2026-406856.pdf]

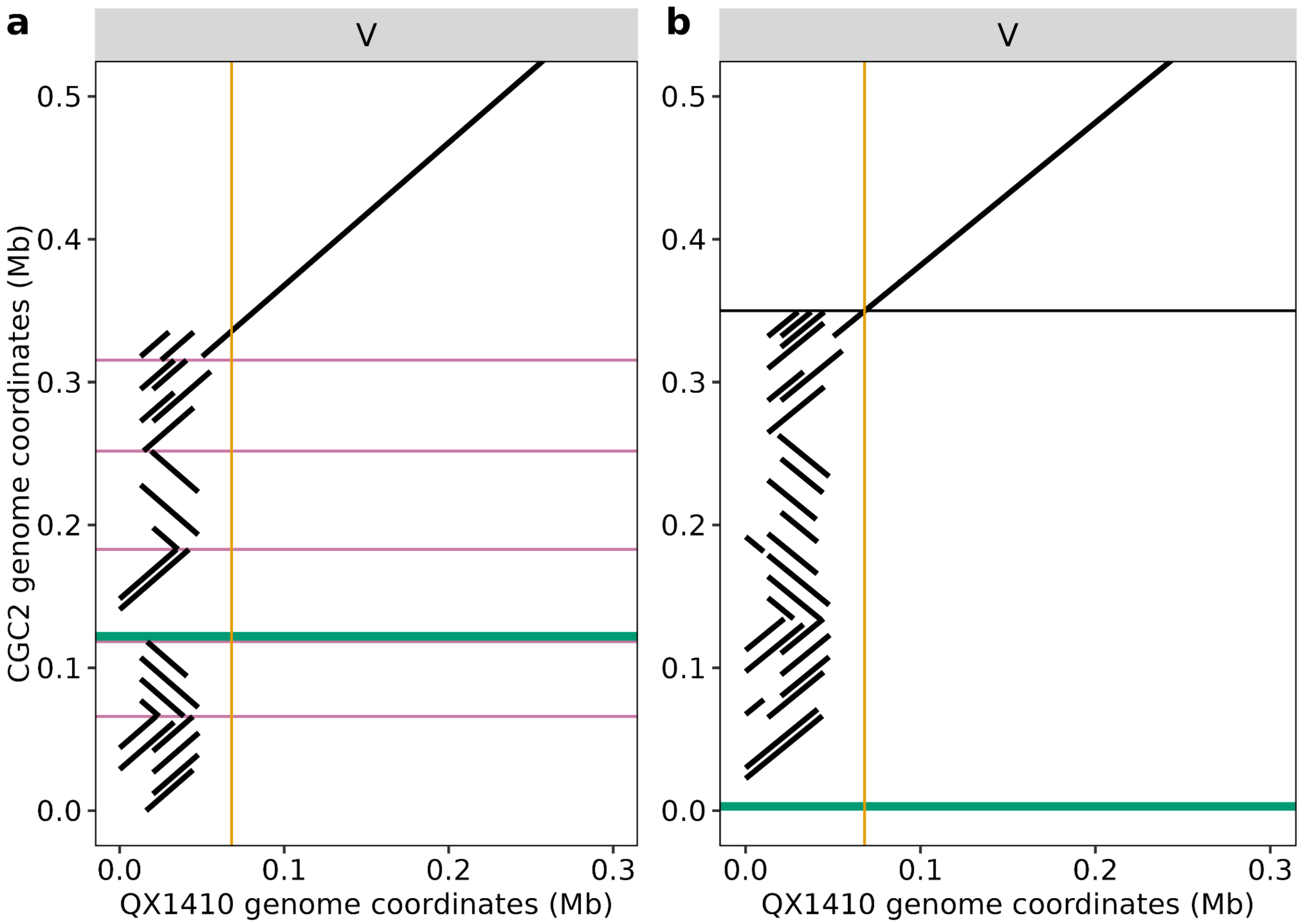

Supplementary Figure 7. Genome-genome alignments of CGC2 to QX1410 highlighting scaffold gaps. (a) Left-end of chromosome V displaying the remaining gaps (horizontal purple lines), telomeric repeat sequence (horizontal green line), and the terminus of rDNA cistron repeats in QX1410 (orange vertical line). (b) The same plot as panel (a) after contig-reordering to place telomeric repeat sequence at the left-end of chromosome V based on Hi-C contact evidence and gap closing using TGS-GapCloser (Xu et al. 2020).
