# Supplementary material for: A gap-free, telomere-to-telomere genome assembly for the Caenorhabditis briggsae reference strain AF16
Source: G3 (Bethesda). 2026 Jun 18;16(8):jkag158. doi: 10.1093/g3journal/jkag158 (PMC13439942; doi:10.1093/g3journal/jkag158)
Supplement: Supplementary file 1 [file jkag158_supplementary_data.zip › Supplementary_Figure_8_G3-2026-406856.pdf]

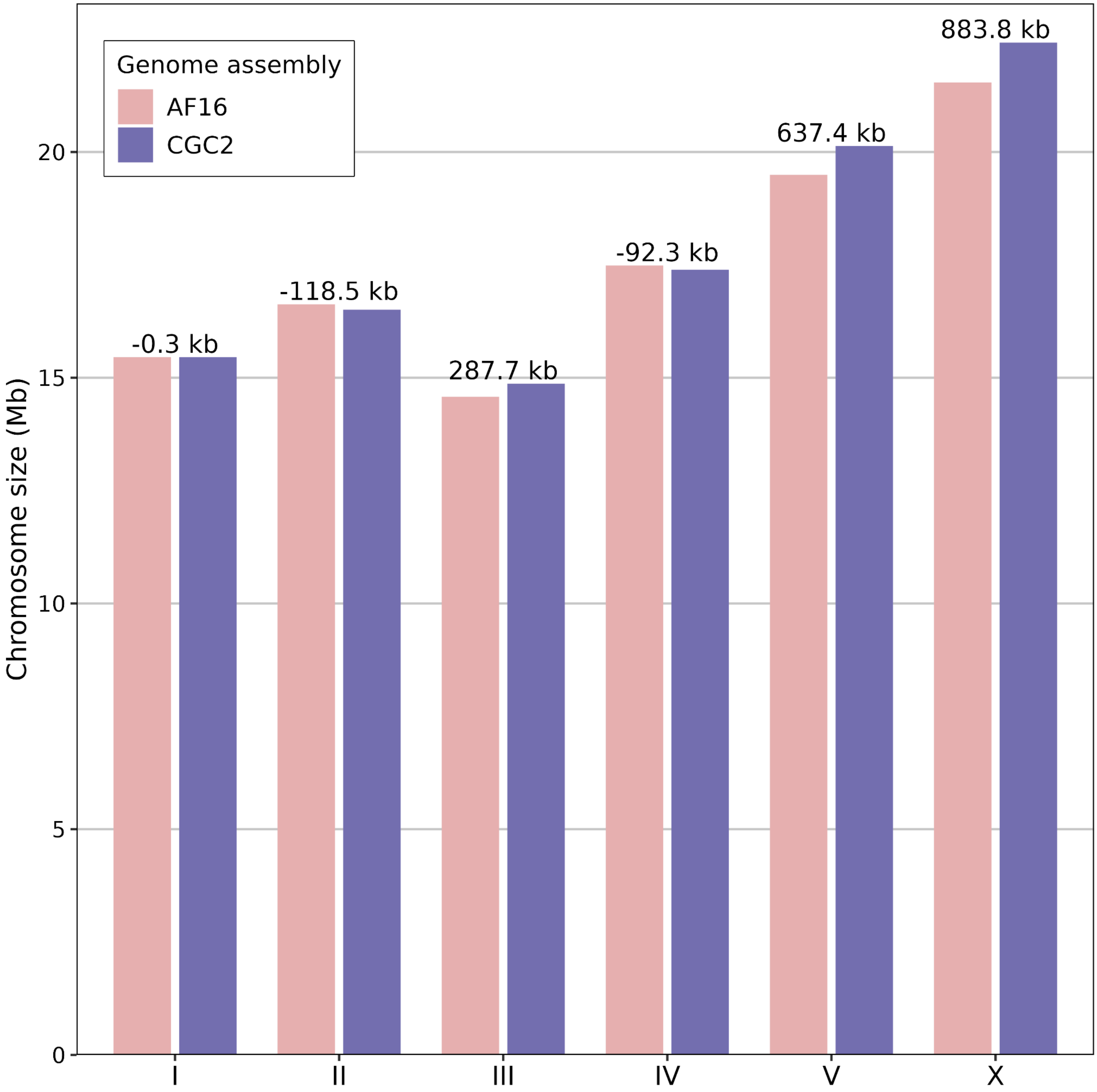

Supplementary Figure 8. Nuclear chromosome sizes of AF16 cb4 and CGC2. Differences in nuclear chromosome sizes of CGC2 compared to AF16 cb4 are displayed above each chromosome set, where the largest difference in span is chromosome X, and smallest difference in span is chromosome I.
