# Supplementary material for: A gap-free, telomere-to-telomere genome assembly for the Caenorhabditis briggsae reference strain AF16
Source: G3 (Bethesda). 2026 Jun 18;16(8):jkag158. doi: 10.1093/g3journal/jkag158 (PMC13439942; doi:10.1093/g3journal/jkag158)
Supplement: Supplementary file 1 [file jkag158_supplementary_data.zip › Supplementary_Figure_9_G3-2026-406856.pdf]

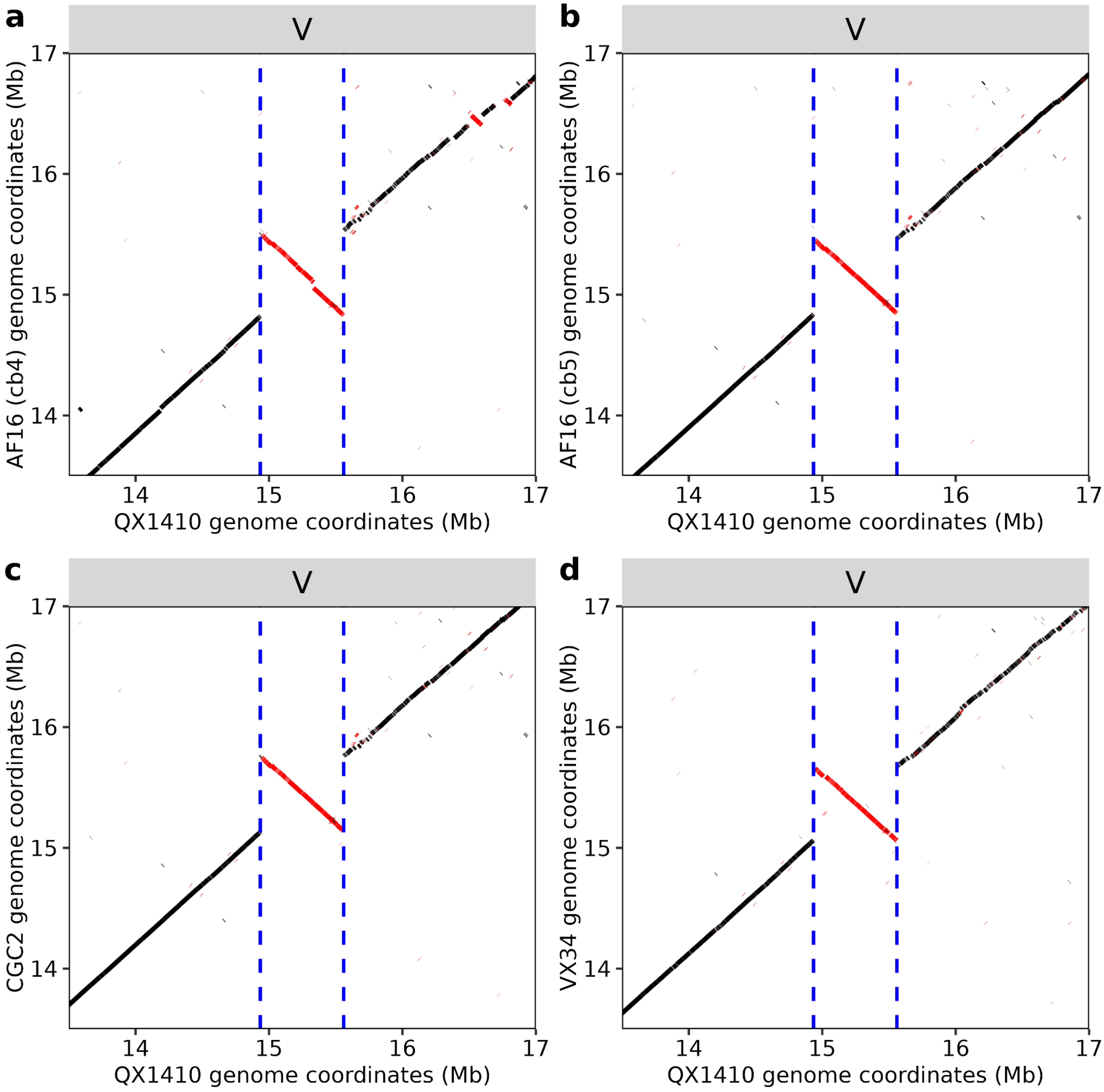

Supplementary Figure 9. A shared inversion in VX34 and AF16 reference genomes on the arm of QX1410 chromosome V. (a) AF16 (cb4) genome alignments to QX1410 highlighting an inversion (red) on the right arm of chromosome V. Vertical blue dashed lines indicate the breakpoints of the inversion in QX1410 genome coordinates. Genome-genome alignments of AF16 (cb5) (b), CGC2 (c), and VX34 (d) to QX1410 highlighting the same inversion (red) found in AF16 (cb4). Vertical blue dashed lines are the same coordinates in all panels.
